# Development and Validation of a Ferroptosis-Related lncRNAs Prognosis Model in Oral Squamous Cell Carcinoma

**DOI:** 10.3389/fgene.2022.847940

**Published:** 2022-03-01

**Authors:** Tao Li, Yi Wang, Xianwang Xiang, Chuanjun Chen

**Affiliations:** ^1^ Department of Oral and Maxillofacial Surgery, The First Affiliated Hospital of USTC, Division of Life Sciences and Medicine, University of Science and Technology of China, Hefei, China; ^2^ WanNan Medical College, Wuhu, China

**Keywords:** ferroptosis, lncRNA, OSCC, immunity, prognosis model

## Abstract

**Objectives:** Ferroptosis is an iron-dependent form of programmed cell death, which affects the prognosis of many cancers. Some long non-coding RNA (lncRNA) can affect the prognosis of cancer by regulating the process of ferroptosis. However, the role of ferroptosis-related lncRNA (frlncRNA) in oral squamous cell carcinoma (OSCC) is not yet clear.

**Materials and Methods:** The data of OSCC patients were downed from The Cancer Genome Atlas (TCGA). After univariate and multivariate Cox regression analysis, the prognosis-related ferroptosis-related lncRNAs were obtained to construct a prognostic model. Calculated the risk score to divide patients into high and low risk groups, and evaluated the predictive ability of the model and the differential expression of immunity in the high and low risk groups.

**Results:** The prognostic model for OSCC was constructed based on 8 prognostic-related frlncRNAs which co-expressed with 25 mRNAs. Kaplan-Meier analyses displayed that the risk score is inversely proportional to patient survival. Receiver operating characteristic (ROC) and decision curve analysis (DCA) indicated that the risk score is superior to other clinical characteristics, and independent prognostic analysis demonstated that risk score is independent factor for the overall survival (OS) rate. The results of immunological analysis showed differences in immune cells, functions, immune checkpoints, and m6A expression between high and low risk groups.

**Conclusion:** We constructed an OSCC patients prognosis model based on 8 frlncRNAs, which can provide prognostic evaluation and immune analysis for OSCC patients, and provided new direction for OSCC targeted therapy.

## Introduction

Oral cancer is one of the common malignant tumors. Recent global estimates show that there will be 377,713 new cases and 177,757 deaths from oral cancer in 2020 (Sung et al., 2021). OSCC is the most common, accounting for more than 90% of all oral cancers (Chi et al., 2015). Although treatment methods are constantly improving, the prognosis of OSCC is still poor, only about 50% in 5 years (Kurihara-Shimomura et al., 2020). At present, the evaluation of prognosis and survival in OSCC is still based on the traditional TNM staging standard. However, due to the differences in the genetic signs of patients with the same TNM staging status, their response to treatment and individual differences may affect the prognosis assessment of patients with OSCC. Therefore, studying the biological, genetic and epigenetic changes of OSCC, especially the underlying mechanism of aggressive phenotype, is essential to improve the prognosis of OSCC patients. Studies have found that programmed cell death (PCD) is related to the occurrence, progression and metastasis of tumors (Lee et al., 2018). Ferroptosis is a new type of PCD, which is different from previous apoptosis and autophagy in its unique mechanism, that is, iron-dependent reactive oxygen species (ROS) accumulation and irresistible lipid metabolism. Oxidation leads to cell death (Dixon et al., 2012). It is well known that inducing cell death is a feasible cancer treatment. Ferroptosis has also been identified as a potential prevention or treatment strategy that triggers cancer cell death, especially for malignant tumors that are resistant to traditional therapies (Roh et al., 2016). Fukuda M et al. reported that ferroptosis plays an important role in oral cancer. Some genes that promote the proliferation of OSCC cells, such as GPX4 and SREBP, seem to protect cells from ferroptosis (Fukuda et al., 2021).

LncRNAs are non-protein coding genes larger than 200 nucleotides to distinguish them from small non-coding RNAs (Kopp and Mendell, 2018). In recent years, with the development of high-throughput sequencing technology, it has been discovered that a large number of non-coding genes play an important role in the occurrence and development of tumors (Gao et al., 2020). Studies have shown that lncRNAs are involved in cell growth, invasion and metastasis. It also plays an important role in OSCC, such as: lncRNACASC9 promotes autophagy apoptosis of OSCC cells by inhibiting the AKT/mTOR signaling pathway to increase autophagy (Yang et al., 2019); lncRNAUCA1 exerts its oncogene effect in OSCC cells through the UCA1/miR-184/SF1 axis (Fang et al., 2017).

**FIGURE 1:**
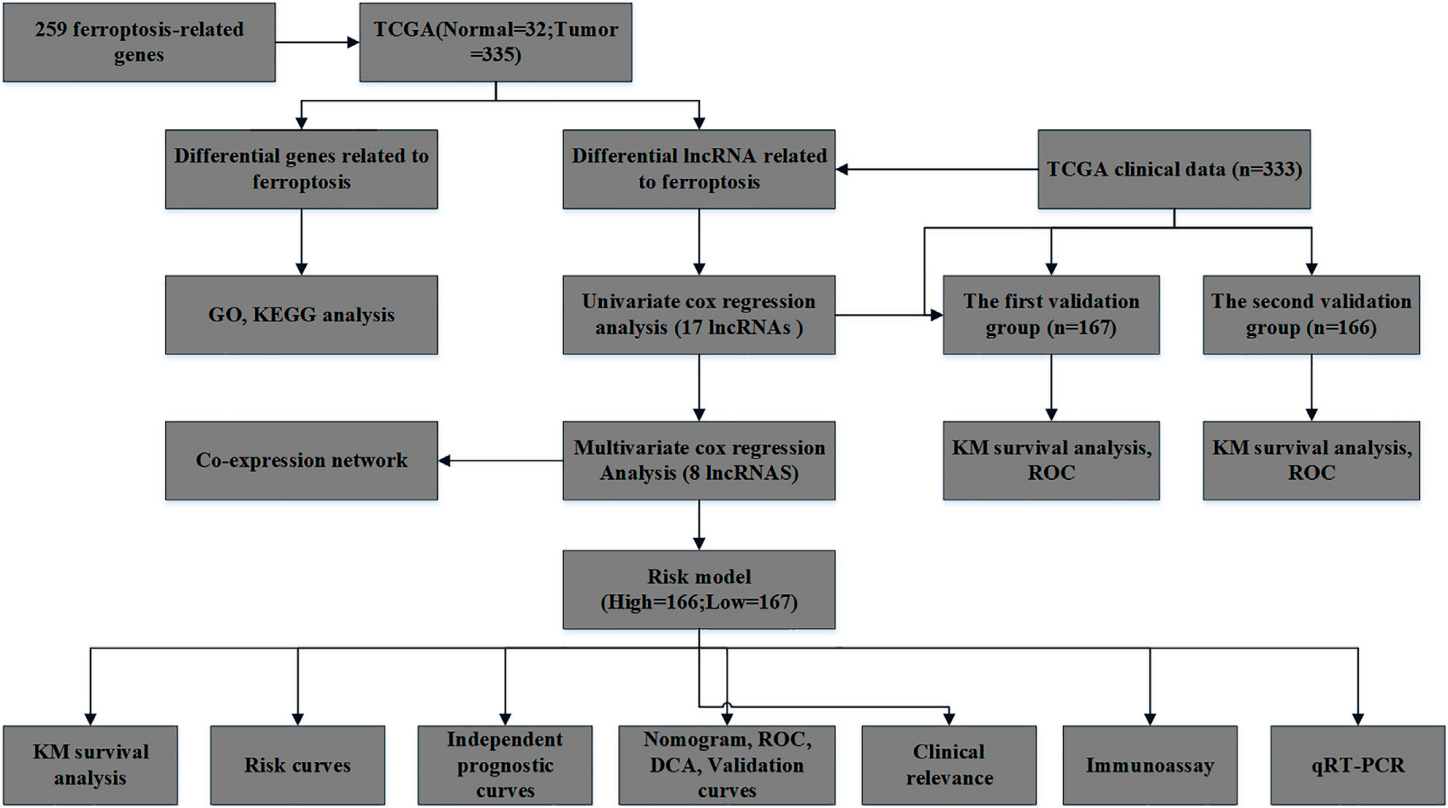
The flowchart of this study.

The role and prognostic value of frlncRNAs in OSCC are currently unclear. Here, we screened frlncRNAs related to the prognosis of OSCC to construct a prognostic model and study its possible mechanism. Meanwhile, we also analyzed the functional enrichment analysis of differential genes, the differences in the expression of immune cell infiltration, immune checkpoint and m6A between high and low risk groups. It is hoped that new biomarkers can be provided for the targeted therapy of OSCC. The flow chart of this study is shown in Figure 1.

## Materials and Methods

### Data acquisition

The RNA sequencing (RNA-seq) data of OSCC patients were downed from TCGA database on 2021.09.29, including 32 samples with normal RNA sequences, 335 samples with OSCC, In addition, we collected 333 cases corresponding clinical datas of tumor samples from TCGA. The clinical data included survival status, survival time, gender, age, tumor stage, pathological grade, T stage, N stage and M stage.

### Identification of frlncRNAs

The human GTF interpretation file were downloaded from Ensembl (http://asia.ensembl.org). 19,573 mRNAs and 14,056 lncRNAs were differentiated and extracted by operating the Strawberry Perl software. A total of 259 ferroptosis-related genes (Supplementary Table S1) included driver genes, suppressor genes and marker genes were extracted from the FerrDb database (http://www.zhounan.org/ferrdb). The correlation between ferroptosis-related genes and all lncRNAs was analyzed through the R limma package to obtain frlncRNAs and ferroptosis-related mRNAs(frmRNAs). The filter standard was set that correlation coefficient >0.4 and *p*-value < 0.001, respectively.

### Screening Differentially Expressed Genes and Enrichment Analysis

The “limma” package of R software was used to screen of differentially expressed genes (DEG) related to ferroptosis between the normal group and the tumor group. The standard is the false discovery rate (FDR) < 0.05 and |logFC| >1. The packages of “colorspace,” “stringi” and “colorspace” were to perform Gene Ontology (GO) analysis and Kyoto Encyclopedia of Genes and Genomes (KEGG) pathway analysis on differentially expressed genes, with *p*-value < 0.05 and *Q*-value < 0.05 as the screening conditions.

### Construction and Evaluation of the Prognostic Model of frlncRNAs

The most prognostic frlncRNAs were screened by univariate and multivariate COX regression analysis. Over and above that, patients were classified into low-risk ( < median) or high-risk ( > median) groups according to the median of risk score. The risk scores of OSCC parents were calculated on the basis of the following formula. N represents the finally optioned lncRNA.

$$\text{Risk}\;\text{score}={\text{Exp}}_{\text{lncRNA}1}\times {\text{β}}_{\text{lncRNA}1}+{\text{Exp}}_{\text{lncRNA}2}\times {\text{β}}_{\text{lncRNA}2}+\cdots +{\text{Exp}}_{\text{lncRNAn}}\times {\text{β}}_{\text{lncRNAn}}$$ (1)

The Kaplan–Meier (K-M) survival curves was used to compare the overall survival (OS) between high-risk and low-risk group, receiver operating characteristic (ROC) curves and decision curve analysis (DCA) were used to evaluate whether the predictive power of risk score was better than that of other clinical characteristics. Univariate and multivariate COX regression analyses were used to determine whether risk scores was independent of other clinical characteristics as a prognostic factor in patients with OSCC. Finally, all independent prognostic parameters were incorporated into the construction of the nomogram to predict the 1, 2, and 3 years overall survival of patients. The accuracy of the nomogram’s predictions was assessed by the calibration curve.

### Internal Validation and Clinical Relevance Analysis

The 333 cliniacal data were randomly divided into two groups to validate the predictive ability of the model according to the ratio of 1:1 through the package “caret” of R (Table 1). In order to compare differences in clinical characteristics between high and low risk groups, the clinical data were divided into several subgroups. Moreover, we also compared the differences in the expression of frlncRNAs in various clinical features.

**TABLE 1:** The clinical datas in different groups.

Variables	TCGA(Total)	Validation	
	(*n* = 333)	The first validation group (*n* = 167)	The second validation group (*n* = 166)
Age (%)			
<=65	210 (63.06)	108 (64.67)	102 (61.45)
>65	123 (36.94)	59 (35.33)	64 (38.55)
Gender (%)			
FEMALE	100 (30.03)	55 (32.93)	45 (27.11)
MALE	233 (69.07)	112 (67.07)	121 (72.89)
Grade (%)			
G1-2	257 (77.18)	127 (76.05)	130 (78.31)
G3-4	67 (20.12)	36 (21.55)	31 (18.67)
GX + unknow	9 (2.70)	4 (2.40)	5 (3.01)
Stage(%)			
StageⅠ-Ⅱ	75 (22.52)	40 (23.95)	35 (21.08)
StageⅢ-Ⅳ	223 (66.97)	108 (64.67)	115 (69.28)
unknow	35 (10.51)	19 (11.38)	16 (9.64)
T (%)			
T1-T2	134 (40.24)	66 (39.52)	68 (40.96)
T3-T4	172 (51.65)	86 (51.50)	86 (51.81)
TX + unknow	27 (8.11)	15 (8.98)	12 (7.23)
M (%)			
M0	121 (36.34)	65 (38.92)	56 (33.73)
MX + unknow	212 (63.66)	102 (61.08)	110 (66.27)
N (%)			
N0	120 (36.04)	63 (37.72)	57 (34.34)
N1-3	158 (47.45)	73 (43.71)	85 (51.20)
NX + unknow	55 (16.51)	31 (18.56)	24 (14.46)

### Establishment of a Co-expression Network of lncRNA-mRNA Related to Ferroptosis

Investigated the relationship between frlncRNAs and frmRNAs, and constructed an lncRNA-mRNA co-expression network through Cytoscape.

### Immunoassay

Immune cell infiltration files were downloaded from (http://timer.cistrome.org).The difference between immune cells in the high and low risk groups were evaluated by the *TIMER, CIBERSORT, CIBERSORT-ABS, QUANTISEQ, MCPCOUNTER, XCELL* and *EPIC*. In addition, we also compared the differences in immune function, immune checkpoint and m6A between high and low risk groups.

### RNA Extraction and Quantitative PCR

There were four pairs of OSCC and adjacent samples collected from the First Affiliated Hospital of University of Science and Technology of China, and stored in liquid nitrogen at -196°C. According to the manufacturer’s instructions, total RNA was extracted by Trizol reagent (Yisheng Biotechnology, China), cDNA was synthesized by HiScript II 1st Strand cDNA Synthesis Kit (+gDNA wiper) (Vazyme, China), Hieff^®^ qPCR SYBR Green Master Mix (Low Rox) (Yisheng Biotechnology, China) was used for amplification, GAPDH was set as an endogenous control. we selected 4 lncRNAs (STARD4-AS1, MIAT, AC099850.3, AL512274.1) of the model for qPCR. The relative quantification method of 2^−ΔΔCT^ was used to normalize the expression of lncRNA. Each group was repeated three times and the mean value was used for analysis. All patients gave informed consent and signed an informed consent form. The primer sequences of these four lncRNAs are provided in Supplementary Table S2.

### Statistical Analysis

All data were analyzed using Rstudio or SPSS 20.0. Paired samples were used by independent *t*-test or one-way analysis of variance. Kaplan-Meier survival analysis was used to assess the difference in survival between the high and low risk groups of OSCC patient prognosis model based on frlncRNAs. ROC and DCA curves were used to evaluate the predictive performance of OSCC prognostic model. The statistical significance was set at *p* < 0.05.

## Results

### Enrichment Analysis of Differential mRNAs Related to Ferroptosis

**FIGURE 2:**
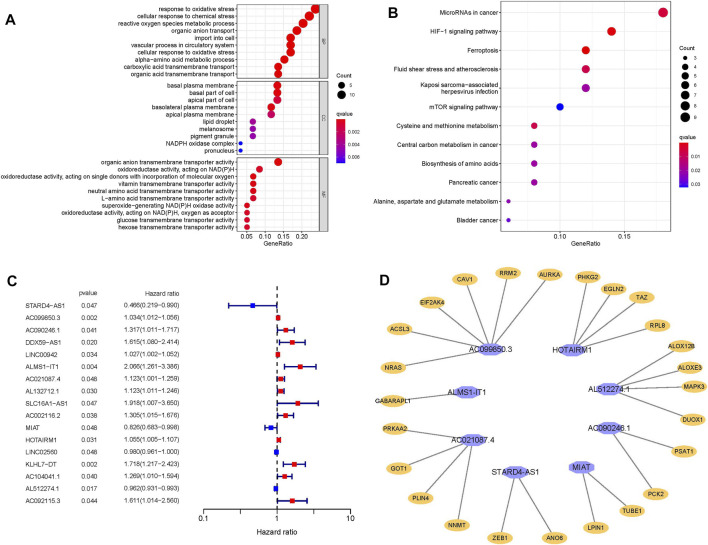
**(A)** Go enrichment analysis. **(B)** KEGG enrichment analysis. **(C)** Forest plot represents 17 lncRNAs related to OSSC prognosis obtained by univariate analysis. **(D)** The prognostic-related lncRNAs and their co-expressed mRNA obtained from multivariate COX regression analysis, purple represents lncRNA, orange represents mRNA.

We found 62 differential mRNAs related to ferroptosis (17 down-regulated and 45 up-regulated) (Supplementary Table S3); BP participated in response to oxidative stress and cellular response to chemical stress; MF regulated organic anion transmembrane transporter activity; CC participated in basal plasma membrane,basal part of cell and apical part of cell (Figure 2A). KEGG analysis results demonstated that overexpressed genes were mainly involved in MicroRNAs in cancer, HIF-1 signaling pathway, Ferroptosis, Fluid shear stress and atherosclerosis, Kaposi sarcoma−associated herpesvirus infection, mTOR signaling pathway, Cysteine and methionine metabolism, Central carbon metabolism in cancer, Biosynthesis of amino acids and Pancreatic cancer (Figure 2B).

### Prognostic-Related frlncRNAs Risk Model and Co-expression Network

A total of 377 differential frlncRNAs were identified, which of 17 were associated with prognosis after univariate analysis (*p* < 0.05) (Supplementary Table S4; Figure 2C). Multivariate COX regression analysis was performed to select the optimal prognostic frlncRNAs, according to Akaike Information Criterion (AIC), finally 8 frlncRNAs were used to construct the OSCC prognostic model (Table 2), and patients were divided into high-risk groups (*n* = 166) and low-risk groups (*n* = 167) based on the median risk score. A total of 25 frmRNAs were co-expressed with these 8 frlncRNAs, of which 6 were co-expressed with AC099850.3 (Supplementary Table S5; Figure 2D).

**TABLE 2:** 8 frlncRNAs by multivariate Cox regression analysis.

LncRNA	Coef	HR	HR.95L	HR.95H	*p-value*
STARD4-AS1	−0.559	0.572	0.270	1.212	0.145
AC099850.3	0.028	1.029	1.007	1.052	0.011
AC090246.1	0.362	1.436	1.051	1.962	0.023
ALMS1-IT1	0.557	1.746	1.034	2.947	0.037
AC021087.4	0.140	1.151	1.027	1.288	0.016
MIAT	−0.238	0.788	0.640	0.970	0.024
HOTAIRM1	0.047	1.050	0.995	1.106	0.079
AL512274.1	−0.038	0.962	0.931	0.996	0.030

### The evaluation of Prognosis Model

**FIGURE 3:**
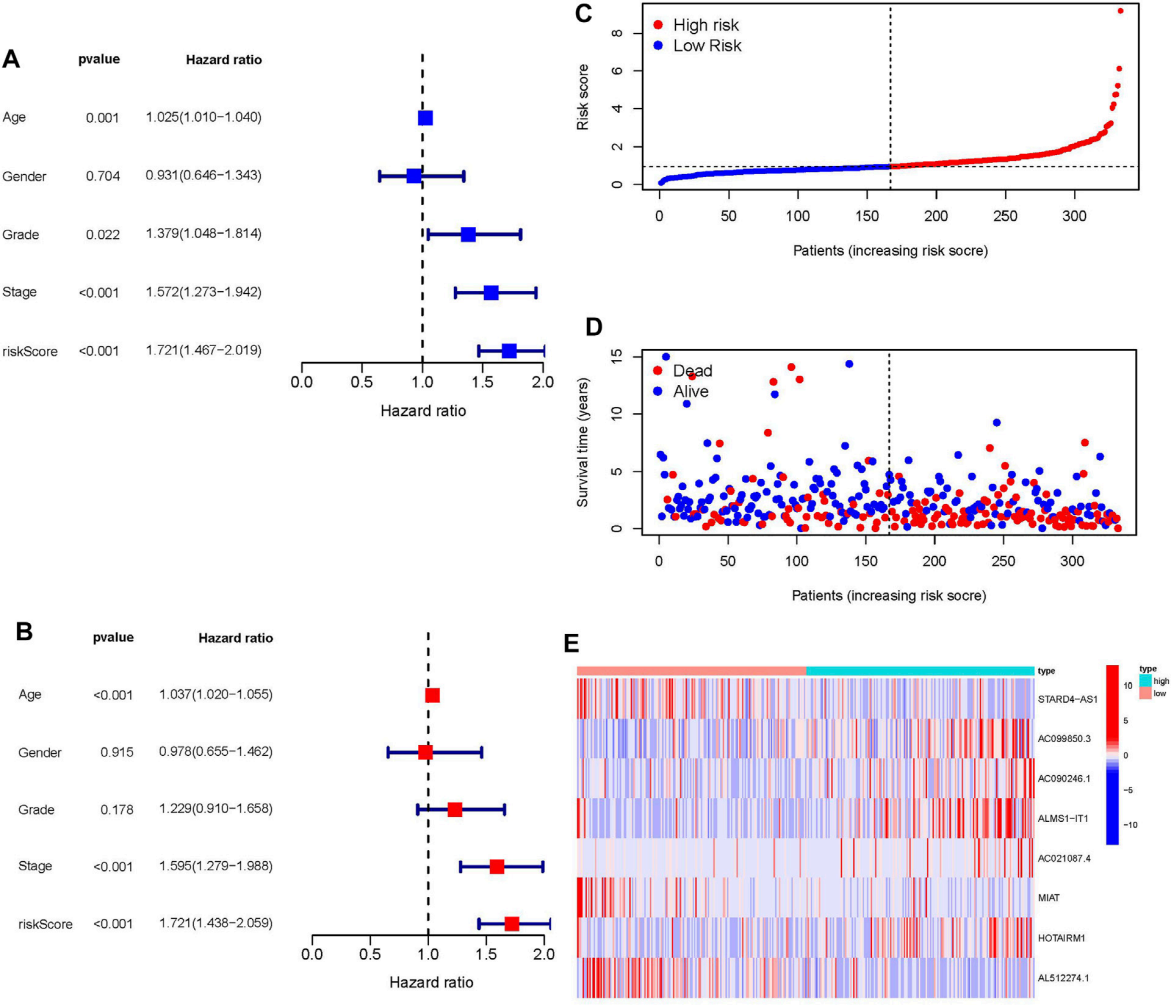
**(A)** Univariate prognostic analysis. **(B)** Multivariate prognostic analysis. **(C)** Risk score of each patient in high-risk ang low-risk group. **(D)** The relationship between patient survival status and risk score. **(E)** Heatmap of frlncRNAs expression with increasing risk score.

**FIGURE 4:**
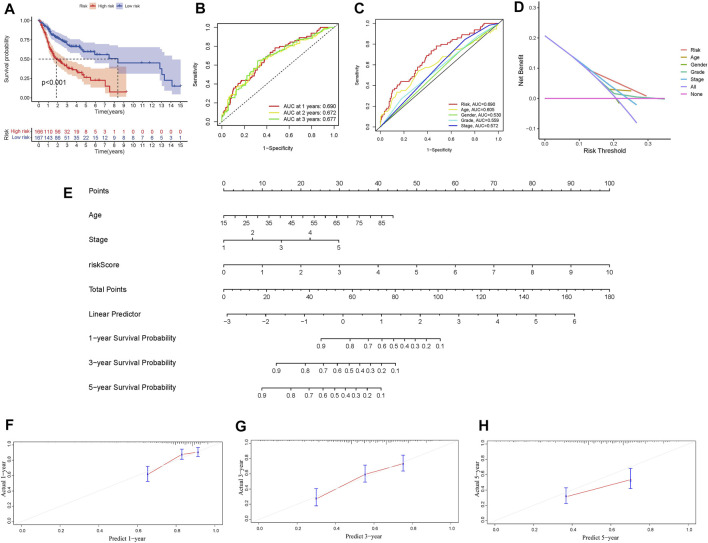
**(A)** Survival analysis of overall survival rate of high and low risk groups. **(B)** Prognosis model 1, 2 and 3 years ROC curve and AUC. **(C)** Comparison of frlncRNAs risk score and other clinicopathological characteristics of AUC. **(D)** DCA curve. **(E)** Nomogram. **(F–G)** Calibration curves for 1year, 3years, and 5 years forecasts of nomogram.

In independent prognostic analysis, the univariate and multivariate COX analysis showed the risk score of frlncRNAs (HR: 1.721, 95CI 1.438−2.059), age (HR: 1.037 95CI: 1.020−1.055) and tumor stage (HR: 1.595, 95CI: 1.279−1.988) were independent prognostic factors of OS in patients with OSCC (*p* < 0.05, Figures 3A,B). There were more deaths can be observed in the high-risk group from Figures 3C,D. We as well observed that STARD4-AS1, MIAT and AL512274.1 were more expressed in the low-risk group; on the contrary, AC099850.3, AC090246.1, ALMS1-IT1, AC021087.4, and HOTARM1 were more expressed in the high-risk group (Figure 3E). In addition, as shown in Figure 4A, Kaplan-Meier analysis illustrated that the expression of high-risk lncRNAs characteristics was significantly different from the low-risk group in OS (*p* < 0.001), and the patients’ risk score was inversely proportional to the survival rate of OSCC patients. It can be seen that there are some numbers under the Figure 4A. For e.g., when the time node is 1, the number corresponding to red is 110, which represents the number of people who survived in the high-risk group when the follow-up time is 1 year. We assessed the sensitivity and specificity of the risk model through AUC (the area under the ROC curve), the AUC of the model to predict 1, 2, and 3 years OS was 0.690, 0.672, and 0.677, respectively (Figure 4B). And the ROC (Figure 4C) and DCA (Figure 4D) curves of the risk score of frlncRNAs indicated that risk score was better than traditional clinical pathology features in predicting the prognosis of OSCC. We included the independent factors of age, tumor stage, and risk score in the multivariate independent prognostic analysis into the nomogram (Figure 4E) to predict 1, 2, and 3 years survival, and used the calibration curve to verify. The results (Figures 4F–H) displayed that the calibration curves were close to the ideal slope, which meaned that age, tumor, and risk scores together can accurately predict 1-, 3-, and 5 years OS of patients.

### The clinical Relevance Analysis and Internal Validation

**FIGURE 5:**
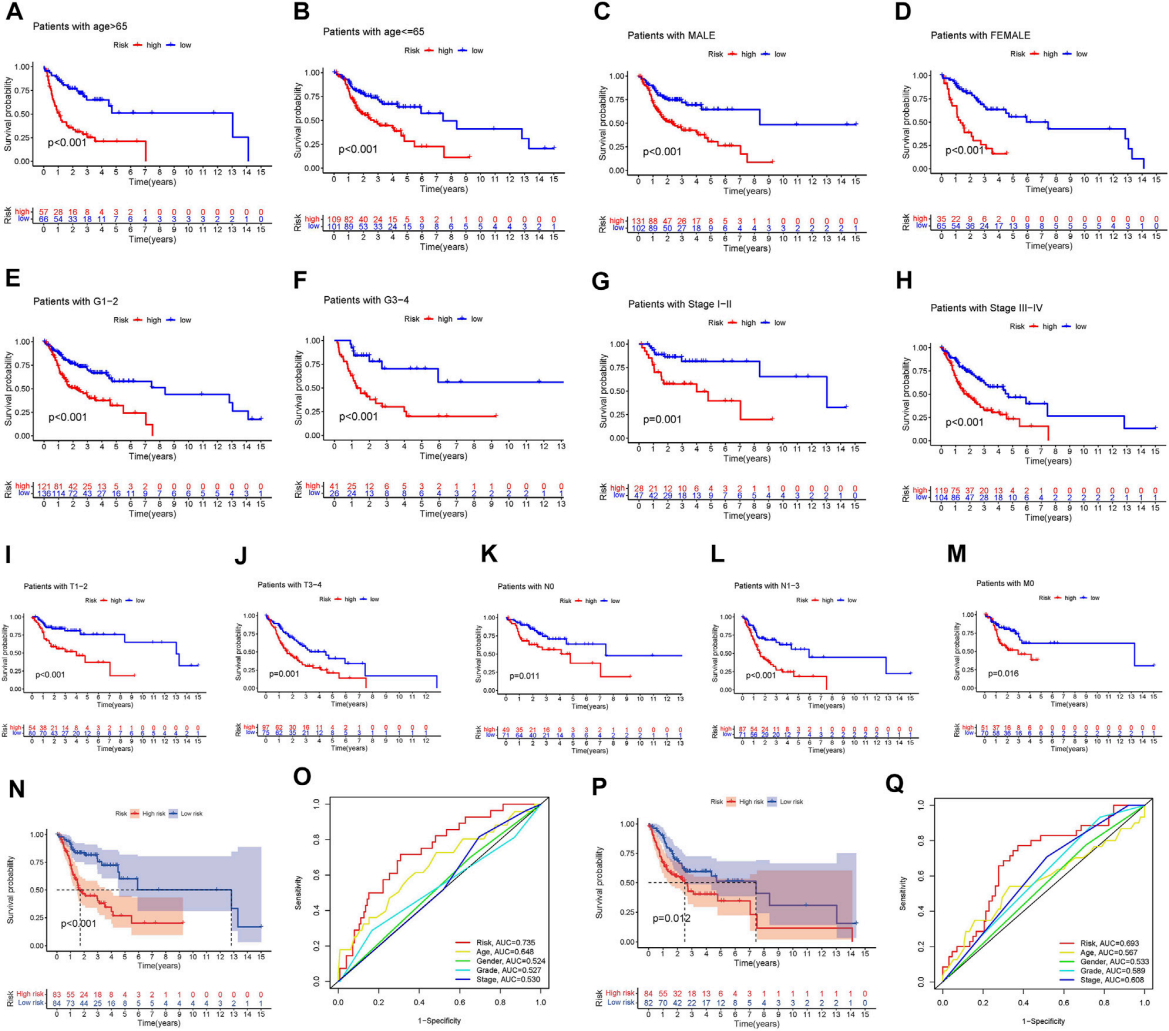
**(A–M)** Kaplan-Meier analysis Survival analysis of high and low groups among various subclinical characteristics. (**A**: age > 65; **B**: age <= 65; **C**: MALE; **D:** FEMALE; **E**: G1-2; **F**: G3-4; **G**: Stage I-II; **H**: Stage III-IV; **I**: T1-2; **J**: T3-4; **K:** N0; **L**: N1-3; **M**: M0) **(N)** Kaplan-Meier analysis of the first validation group. **(O)** Comparison of the predictive ability of the risk score with other clinical characteristics in the first validation group. **(P)** Kaplan-Meier analysis of the second validation group. **(Q)** Comparison of the predictive ability of the risk score with other clinical characteristics in the first validation group.

**FIGURE 6:**
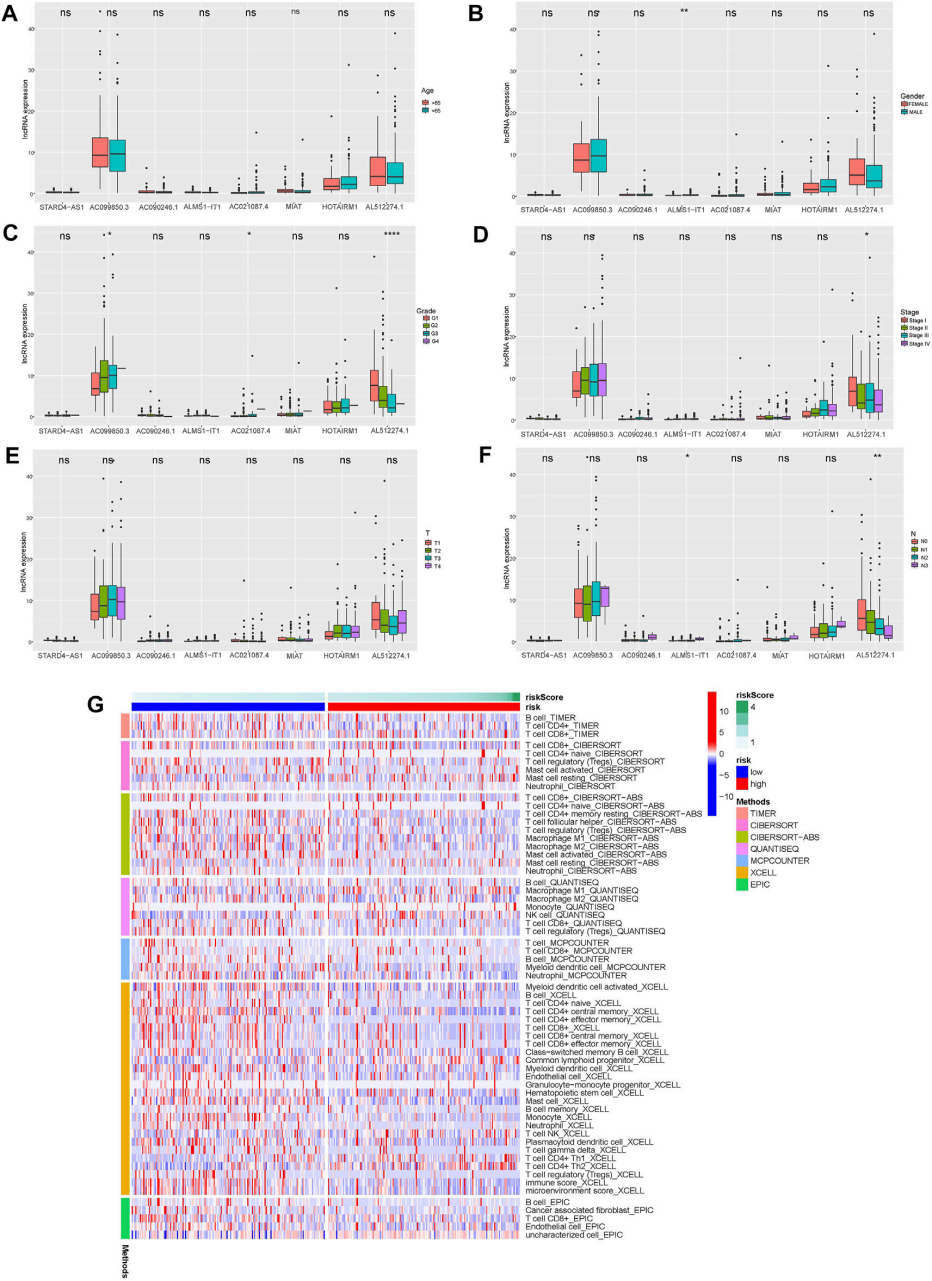
**(A–F)** Differences in the expression of 8 frlncRNAs among clinical variables (**A**: age; **B**: Gender; **C**: Grade; **D**: Stage; **E**: T stage; **F**: N stage; *p < 0.05; **p < 0.01; ***p < 0.001; ****p < 0.001). **G**: Heat map of immune cell expression in high and low risk groups.

The clinical variables were divided into several subgroups. We compared the differences in the OS of patients in the high and low risk groups in various clinical variables through Kaplan-Meier analysis. It can be seen that the OS of patients in the high risk group is lower in all subgroups including (age, gender, grade, stage, Tstage, N stage, M stage; *p* < 0.5, Figures 5A–M). Furthermore, The results of the Kaplan-Meier analysis of the first validation group and the second validation group illustrated that the OS of the high-risk group was lower (*p* < 0.5, Figures 5N,P), and the AUC of the risk score in both groups was greater than that of other clinical characteristics, which indicated that the predictive performance of the risk model was good (Figures 5O,Q). We also analyzed the differences in the expression of frlncRNAs among different clinical features(Figures 6A–F). It can be observed that ALMS1-IT1 was differentially expressed in males and females (Figure 6B). In addition, its expression is differential in N stage (Figure 6D); AL512274.1 had differences in the expression of grade, stage, and N stage (Figures 6C,D,F); AC099850.3 was differentially expressed on different grades (Figure 6C).

### Immunity and Gene Expression and qPCR

**FIGURE 7:**
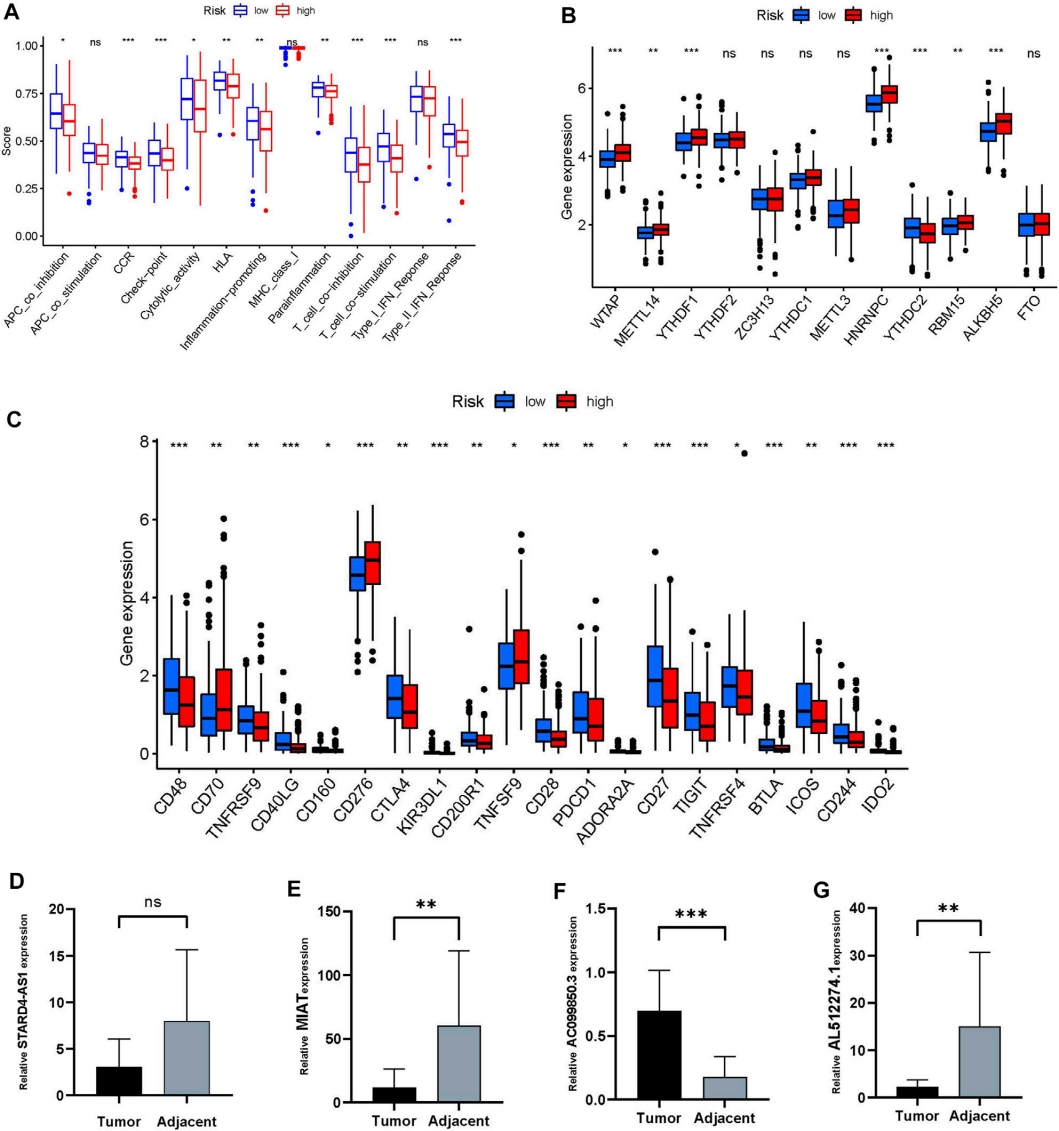
Differences in immune status between high and low risk groups. **(A)** immune function. **(B)** Differences in m6A expression between high and low risk groups. **(C)** Differences in immune checkpoints between the two groups. **(D–G)** The relative expression of four frlncRNAs (STARD4-AS1, MIAT, AC099850.3, AL512274.1) between tumor and adjacent tissue (*p < 0.05;**p < 0.01; ***p < 0.001)

The immune response heatmap based on *TIMER, CIBERSORT, CIBERSORT-ABS, QUANTISEQ, MCPCOUNTER, XCELL*, and *EPIC* algorithms is shown in the Figure 6G. Analysis of differences in immune function based on TCGA-OSCC data showed that APC_co_inhibition, CCR, Check-point, Cytolytic_activity, HLA, Inflammation-promoting, Parainflammation, T_cell_co-inhibition, T_cell_co-stimulation and Type_II_IFN_Reponse were all highly expressed in the low-risk group (Figure 7A). At the same time, the comparison of m6A-related gene expression in high and low groups suggested that the expressions of WTAP, METTL14, YTHDF1, HNRNPC, YTHDC2, RBM15 and ALKBH5 were different (*p* < 0.05, Figure 7B). In view of the importance of checkpoint inhibitor-based immunotherapy, we further explored the differences in immune checkpoint expression between the two groups. The results demonstrated that between the two groups *CD48*, *TNFRSF9*, *CD40LG*, *CD160*, *CTLA4*, *KIR3DL1, CD200R1, CD28, PDCD1*, *ADORA2A*, *CD27*, *TIGIT*, *TNFRSF4*, *BTLA*, *ICOS*, *CD244*, and *IDO2* were expressed higher in the low-risk group;*CD70, CD276, TNFSF9* were the opposite (*p* < 0.05, Figure 7C). As shown in Figures 7D–G, MIAT, AL512274.1 and STARD4-AS1 were more highly expressed in adjacent tissues than tumor, and AC099850.3 was highly expressed in tumor tissues, which is consistent with our model.

## Discussion

Ferroptosis is an iron-dependent cell death program, which has been shown to be related to tumor development and response to anti-tumor therapy (Chen et al., 2021a). LncRNA is an active participant in the immune regulation of 33 cancer types (Li et al., 2020a). Morever, many lncRNAs are involved in the progression of malignant tumors and tumor resistance, and have become new biomarkers and therapeutic targets in cancer diagnosis and treatment (Wang et al., 2019a). The excellent predictive ability of frlncRNAs in cancer has been confirmed in a variety of cancers (Chen et al., 2021b; Lu et al., 2021; Jin et al., 2021; Li et al., 2021). However, the mechanism of frlncRNAs in OSCC is still unclear. Therefore, we conducted research on the correlation between frlncRNAs and the prognosis of OSSC. Based on 8 frlncRNAs, we constructed an OSSC risk prognosis model and divided patients into high and low risk groups according to risk scores. ROC, DCA, independent prognostic analysis to verify its predictive ability, the results showed that its predictive ability is better than other clinical features. These 8 frlncRNAs contained 5 risk factors: AC099850.3, AC090246.1, ALMS1-IT1, AC021087.4, and HOTARM1; three protection factors: STARD4-AS1, MIAT and AL512274.1. Among them, AC099850.3 is the most co-expressed lncRNA, which is related to six differently expressed mRNAs, namely (CAV1, NRAS, ACSL3, AURKA, EIF2AK4 and RRM2), and its high expression level is closely related to the reduction in the survival rate of patients with tongue cancer (Zhou et al., 2019). The high expression of ALMS1-IT1 can lead to poor prognosis of many cancers, such as head and neck squamous cell carcinoma (Xing et al., 2019), small cell lung cancer (Luan et al., 2021). The mechanism of its regulation is not yet fully understood. There are studies have shown that in small cell lung cancer, ALMS1-IT1 regulates AVL9 by adsorbing miRNAs, and participates in the regulation of cell cycle-related CDK pathways, thereby affecting tumor progression (Luan et al., 2021). HOTAIRM1 plays different roles in different diseases. It promotes autophagy and proliferation of acute myeloid leukemia cells with mutant nucleophosphoprotein by regulating the expression of EGR1 and ULK3 (Jing et al., 2021). HOTAIRM1 is down-regulated in liver cancer. The specific mechanism may be related to inhibiting the Wnt pathway to inhibit the proliferation of hepatocellular carcinoma cells and promote their apoptosis, thereby inhibiting the progression of liver cancer (Zhang et al., 2018). It is worth noting that as an oncogene, MIAT can proliferate and migrate in various cancer cells such as hepatocellular carcinoma (Huang et al., 2018), osteosarcoma (Zhang et al., 2019), and papillary thyroid carcinoma (Wang et al., 2019b). However, in our study, single-factor and multi-factor COX regression analysis showed that MIAT is a protective factor for the prognosis of OSCC. Furthermore, as shown in Figure 7E, MIAT expressed higher in adjacent tissues, which is consistent with our model. The role of AL512274.1 in cancer is still unclear, but studies have found that its co-expressed mRNA (MAPK3) is involved in the control of cell proliferation, differentiation and autophagy (Cagnol and Chambard, 2010; Jiang et al., 2021). STARD4-AS1, AC090246.1, AC021087.4 have not seen relevant studies in oral cancer and other tumors, and the specific mechanism is worthy of further investigation.

Considering that immunotherapy is playing an increasingly important role in cancer treatment. We compared the differences in immunity between high and low risk groups. As the Figure 7C displays that, except for CD70, CD276 and TNFSF9, almost all other differential genes in the immune checkpoint were expressed in the low-risk group, while m6A except YTHDC2 were mainly expressed in the high-risk group. Studies have reported that CD70 is highly expressed in oral cancer, and its specific CAR-T cells can specifically recognize and effectively eliminate CD70-positive HNSCC cells (Park et al., 2018). In addition, CD276 is highly expressed in head and neck squamous cell carcinoma and the blockade of CD276 significantly inhibited the lymph node metastasis of head and neck squamous cell carcinoma, which is one of the most challenging problems in the treatment of head and neck squamous cell carcinoma, indicating that targeting CD276 can enhance anti-tumor immunity (Wang et al., 2021). There are no studies on TNFSF9 and YTHDC2 in oral cancer. Yang Li reported that YTHDC2 is a tumor suppressor gene in the head and neck, which is highly expressed in normal tissues but low in tumors (Li et al., 2020b). In general, frlncRNAs have shown excellent performance in our research and are expected to become new biomarkers for the treatment of OSCC. However, there are some deficiencies in this study. First, the establishment and validation of the risk model is based on the TCGA database and its grouping, and there is a lack of external validation to provide more evidence for evaluating its clinical utility. Second, the number of experimental validations is limited, and the role and mechanism of most frlncRNAs in the progression of OSCC in this study are not clear, and further investigation is needed.

## Conclusion

We constructed an OSCC patients prognosis model based on 8 frlncRNAs, which can provide prognostic evaluation and immune analysis for OSCC patients, and provided new direction for OSCC targeted therapy.

## Data Availability

The original contributions presented in the study are included in the article/Supplementary Material, further inquiries can be directed to the corresponding author.
